# Supplementary material for: Validation of saline, PBS and a locally produced VTM at varying storage conditions to detect the SARS-CoV-2 virus by qRT-PCR
Source: PLoS One. 2023 Feb 13;18(2):e0280685. doi: 10.1371/journal.pone.0280685 (PMC9924993; doi:10.1371/journal.pone.0280685)
Supplement: S2 Table — (DOCX) [file pone.0280685.s002.docx]

**Supplementary Table 2.**

|  | Temperature | | | |  | |
| --- | --- | --- | --- | --- | --- | --- |
| Media | -80°C | 4°C | Room  temperature | 37°C | | Total |
| SUTM | 4 | 1 | 0 | 0 | | 5 |
| RVTM | 2 | 2 | 1 | 0 | | 5 |
| PBS | 2 | 2 | 0 | 0 | | 4 |
| Normal Saline | 2 | 0 | 0 | 0 | | 2 |
| Total | 10 | 5 | 1 | 0 | | 16 |
